# Navigating the crisis landscape: engaging the ministry of health and United Nations agencies to make abortion care available to Rohingya refugees

**DOI:** 10.1186/s13031-020-00298-6

**Published:** 2020-07-23

**Authors:** Tamara Fetters, Sayed Rubayet, Sharmin Sultana, Shamila Nahar, Shadie Tofigh, Lea Jones, Ghazaleh Samandari, Bill Powell

**Affiliations:** 1 Ipas, Chapel Hill, North Carolina USA; 2 Ipas Bangladesh, Dhaka, Bangladesh; 3 Public Health Leadership Program, Chapel Hill, North Carolina USA

**Keywords:** Abortion, Menstrual regulation, Unintended pregnancy, Refugees, Sexual violence, Rohingya, Bangladesh

## Abstract

**Background:**

Unintended and unwanted pregnancies likely increase during displacement, making the need for sexual and reproductive health (SRH) services, especially safe abortion, even greater. Attention is growing around barriers to safe abortion care for displaced women as donor, non-governmental and civil society actors become more convinced of this need and reports of systematic sexual violence against women are more widely documented around the world. Yet a reluctance to truly change practice remains tied to some commonly reported reasons: 1) There is no need; 2) Abortion is illegal in the setting; 3) Donors do not fund abortion services, and; 4) Abortion is too complicated during acute emergencies. While there is global progress towards acknowledging the deficit of attention and evidence on abortion services in humanitarian settings, improvements in actual services have yet to follow.

**Case presentation:**

In August 2017, over 700,000 Rohingya refugees fled Myanmar for Bangladesh. Women and girls fled homes and communities - many experienced terrible violence - and arrived at camps in Bangladesh with SRH needs, including unwanted pregnancies. With funding from UNFPA and others, Ipas trained providers and established safe induced abortion (called menstrual regulation (MR) in Bangladesh) and contraception services in October 2017.

Ipas Bangladesh initiated the trainings in coordination with the government’s health system and international aid agencies. Training approaches were modified so that providers could be trained quickly with minimal disruption to their ability to provide care. Within one month of the arrival of refugees, MR services had been established in eight facilities, for the first time during an acute emergency. By mid-2019, over 300 health workers from 37 health facilities had attended training in MR, postabortion care (PAC), and contraception. Over 8000 Rohingya refugees have received abortion-related care, more than three-quarters of which were MR procedures; over 26,000 women and girls have received contraception at these facilities.

**Conclusions:**

This study demonstrates demand for abortion care exists among refugees. It also illustrates that these needs could have been easily overlooked in the complex environment of competing priorities during an emergency. When safe abortion services were made available, with relative ease and institutional support, women sought assistance, saving them from complications of unsafe abortions.

## Introduction

In 2017, an eruption of violence in Myanmar sent nearly 650,000 Rohingya refugees across the border into to Bangladesh. The influx of refugees was among the fastest the world had ever seen, occurring primarily over 2 weeks beginning August 25, 2017, after militants launched deadly ethnic cleansing in the Rakhine state [1]. During crises such as this one, host countries, United Nations (UN) agencies, and national and international non-governmental organizations (NGOs) join forces to provide protection, food security and health care to the best of their abilities. In addition to the need for basic food, shelter, and security, women face unique hardships trying to prevent unwanted pregnancy due to lost livelihoods, disrupted family structures, exposure to sexual violence and interrupted access to sexual and reproductive health (SRH) services [2–5].

Unsafe abortion occurs everywhere, but 97% of all cases occur in fragile or limited resource countries [6]. Sexual violence in conflict zones is multi-faceted and can include rape, sexual slavery, forced prostitution, forced pregnancy, forced abortion, trafficking and forced marriage, imposing fear and intimidation in times of conflict [7]. As a result, unintended and unwanted pregnancies likely increase during displacement making the need for SRH services, especially safe abortion, even greater.

Efforts to secure essential SRH services in humanitarian crises can be chaotic. It is often not prioritized or recognized as a component of protection and essential life-saving health interventions. The humanitarian community continues to struggle with how best to provide these services as quickly and efficiently as possible. This case study describes the first introduction of all life-saving SRH services, including safe and legal abortion services during an acute emergency in Bangladesh. This case study delves into the historical obstacles to providing safe abortion during crises and makes the case for improving SRH programming in all humanitarian settings and provides evidence to show that, with leadership and prioritization, safe abortion care can be introduced in the early days of an emergency.

## Historical lack of safe abortion services in humanitarian settings

Globally, efforts to introduce abortion care in the first months of an emergency have been erratic and piecemeal at best. Only one major humanitarian organization, Médecins Sans Frontières (MSF), openly broadcast their efforts to provide lifesaving safe abortion services in humanitarian settings, without regard to national laws in the countries where they work. MSF published their 2004 organizational policy decision stating that this was their obligation and necessary to reduce maternal mortality and suffering and prevent unsafe abortions in the countries where they work [8]. Initially, there was resistance to the policy change within the organization, so MSF began an international effort to help staff examine values and attitudes about abortion, along with clinical training and roll-out of their safe abortion initiative [9].

A global evaluation of SRH service provision in humanitarian settings conducted in 2013, confirmed that safe abortion was unavailable in all 63 facilities serving displaced people that were assessed [10]. In 2015, a global review of grant applications found that contraception, especially emergency and long-acting contraception, as well as safe abortion were significantly under-represented in humanitarian appeals [11]. A 2016 article by researchers at the Columbia University Mailman School of Public Health explored humanitarian organization capacity and attitudes about abortion care and presented their findings. In that work, the authors confirmed that agencies play a powerful role in restricting women’s access to safe abortion and contraceptive care in crisis settings believing that abortion was not provided because: 1) There is no need; 2) Abortion is illegal in the setting; 3) Donors do not fund abortion services, and; 4) Abortion is too complicated to provide during acute emergencies [12]. The experience in Bangladesh documented here disproves these beliefs as fallacies.

Over the past decade, as donors became more convinced about the need for earlier and more comprehensive SRH services during crises, many stepped in to promote better documentation and address the evidence gap around abortion in humanitarian settings, as a key component that is often excluded. As for illegality, only 26 countries in the world ban abortion entirely [13]. Even among countries that severely restrict abortion, 46 of them allow abortion in cases of rape [14]. Additionally, international humanitarian law and agreements such as the Geneva Convention, UN Security Council resolutions and the Maputo Protocol strongly support access to safe abortion for survivors of rape [15]. A groundswell of new attention has begun to grow around the reluctance to provide safe abortion care to displaced women as donor, NGO and civil society actors have become more convinced of this need and reports of systematic sexual violence against women have become more widely documented around the world. While there is global progress towards acknowledging the deficit of attention and evidence on abortion services in humanitarian settings, improvements in actual services have yet to follow [15, 16].

## Resistance to abortion provision in humanitarian crises

Although the broader humanitarian community now largely agrees that providing SRH to refugees is important, the issue of when to introduce abortion remains a topic of debate among humanitarian health care practitioners, primarily because of concerns about the perceived complexity of introducing abortion as a priority intervention during an acute emergency. In fact, with proper training, early abortion is among the simplest and most common medical procedures. Abortion is safer than childbirth [17]. A maternal death due to an abortion in the US is as rare as a death due to a shot of penicillin, less than 1 per 100,000 procedures [18]. Additionally, since many pregnancies result in miscarriages, physicians and many nurse-midwives, are taught to perform uterine evacuations for these procedures during their medical educations. The ability to evacuate a uterus is recognized as a life-saving and essential signal function for basic and comprehensive emergency obstetric and neonatal care. This same procedure is also used to perform an induced abortion. With the recent introduction of mifepristone into the global humanitarian supply chain of the United Nations Population Fund (UNFPA), an abortion procedure with medication is even less invasive and can be, for the most part, completed by a woman in private. Misoprostol, which can be used alone or in conjunction with mifepristone to induce a medical abortion, is already available in labor and delivery and PAC kits to treat complications of an unsafe abortion or miscarriage in many humanitarian health facilities. Misoprostol is also widely available in the markets and pharmacies of most countries.

The Inter-Agency Working Group on Reproductive Health in Crisis (IAWG) came together over two decades ago to bring attention to the neglected issue of SRH in conflict and disaster settings. At first efforts focused on the need to provide basic SRH services. The IAWG was instrumental in developing a seminal document for SRH technical updates called the Inter-Agency Field Manual (IAFM) [19, 20], now revised and published 3 times. In 2010, partners in the IAWG agreed that it was time to include a chapter on safe abortion in humanitarian settings. In the recent 2018 revision, the chapter was upgraded for broader inclusion of safe abortion care throughout the 2018 IAFM. Most technical experts involved in the nearly two years of revisions felt that withholding information on abortion would be a political rather than a technical decision and opted to leave it in the manual. Ultimately, after lengthy debates among the IAWG members, which includes multiple United Nations representatives, abortion was included as an “additional priority activity” and included in the newest version of the Minimum Initial Service Package (MISP), a coordinated set of priority activities designed to prevent excess morbidity and mortality, particularly among women and girls at the onset of humanitarian emergencies that should be implemented at the onset of every humanitarian emergency.

However, not all in the humanitarian community agreed. In their 2018 article on the 2018 IAFM revision [21], the authors describe feeling that the process of including safe abortion care in the MISP had gone too far. In an argument that echoes, “abortion is too complicated” in the early days of an emergency, Tran and Schulte-Hillen contend that during acute emergencies, the technical capacity and leadership on SRH is too limited to attempt to develop and implement abortion services.

## Discussion: considerations for introducing and scaling up safe abortion during an acute emergency

### Advocating for SRH during the Rohingya refugee crisis

Soon after Rohingya started fleeing Myanmar, the United Nations organizations quickly expanded their local presence and joined the government of Bangladesh in the planning and implementation of services for the new refugee population. In adherence with the Sphere model to improve the minimum standards for quality of humanitarian assistance during an emergency, international stakeholders began to assess and act to improve minimum standards in the four primary life-saving areas of humanitarian aid: water supply, sanitation and hygiene promotion; food security and nutrition; shelter, settlement and non-food items; and health action [22]. Within months, with the World Health Organization leading coordination, the national health cluster quickly grew to more than 120 national and international partners assisting with the provision of health services in 169 health facilities in 30 refugee camps in the Cox’s Bazar district, a coastal location in southeastern Bangladesh bordering Myanmar.

Technical experts in SRH at UNFPA called together the first meeting of the Technical Working Group for SRH in Cox’s Bazar to plan for addressing the SRH needs of the Rohingya refugees in the first week of September 2017. A key component of the working group was to plan for the introduction and coordination of the MISP for reproductive health as outlined in the IAFM [20]. The MISP is not considered one of the core life-saving areas of Sphere but, according to the Inter-Agency Field Manual on Reproductive Health in Humanitarian settings, it is an SRH priority meant to be introduced as early as is feasible during an emergency [20].

### The role of Ipas in safe abortion provision in Bangladesh

In Bangladesh, abortion is restricted except to save the life of a woman, but menstrual regulation (MR) has been part of Bangladesh’s national family planning program since 1979. MR is a procedure that uses manual vacuum aspiration (MVA) or a combination of mifepristone and misoprostol to “regulate the menstrual cycle when menstruation is absent for a short duration” [23]. Ipas is an international nongovernmental organization (NGO) dedicated to ending preventable deaths and disabilities from unsafe abortion and to preventing unintended pregnancy by providing access to contraception and comprehensive abortion care. Since 2011, the Ipas Bangladesh country program has supported the Ministry of Health and Family Welfare to develop and increase women’s access to high-quality integrated contraception, MR, and PAC services in the public sector. Ipas works with a wide range of stakeholders within Bangladesh to train abortion providers, connect women with vital information so they can access services, and to advocate for safe, legal abortion.

### Assessing the need for and status of abortion among Rohingya refugees

On September 25, 2017, at the request of the UNFPA, staff from Ipas Bangladesh joined representatives from around the nation in the SRH technical working group to discuss and plan for the SRH service assessment, monitoring, scale-up and implementation of SRH services for Rohingya refugees. Upon discovering that MR was being neglected in the case of Rohingya women despite being a national policy, Ipas staff set out to informally assess SRH and the availability of MR throughout Cox’s Bazar. After visiting several health facilities and interviewing providers in the area, the Ipas team discerned that MR was available in only one district-level health facility serving a population of over 3 million people. Additionally, none of the health facilities in the area were able to provide long-acting or reversible contraception, like intrauterine devices and implants.

Restrictions on funding for MVA and training for induced abortion care (due to United States policies such as the Helms Amendment and the Protecting Life in Global Health Assistance (PLGHA) policy) limited the availability of service providers for training, as Bangladeshi employees working for US-funded NGOs were unwilling or unable to attend trainings, even though MR services are legal in Bangladesh [24, 25]. United States policies did not prohibit funding for contraceptive services, including emergency contraception, PAC or the legal indications for induced abortion allowed under the PLGHA, that is for cases of rape, incest or to save the life of the woman [25]. However, prioritization of these services early in the crisis was extremely limited or nonexistent. During the initial period, stakeholders also cited several additional reasons for not introducing abortion services to the refugee community such as perceptions that MR would not be supported by Rohingyas, who are known to be religiously conservative, and beliefs that Rohingya families desired more children so there would be no demand for abortion in this population. Recruitment of abortion service providers was further complicated because of their beliefs that the service was not needed.

Despite initial obstacles, the policy environment for MR in Bangladesh provided a basis for exploring the inclusion of MR for refugees. The Ipas team delved deeper into the supply-side perceptions about abortion provision for Rohingya through interviews with providers and stakeholders and found that the first challenge was in the way Bangladeshi providers defined “comprehensive SRH”. For many local providers, “comprehensive SRH” did not include abortion, even for survivors of sexual assault. Furthermore, the idea that Rohingya women did not want MR was refuted through informal interviews with refugees in the hospital waiting areas during the assessment, many of whom acknowledged the danger and burden of high fertility. Finally, in the refugee camps, Ipas noted high numbers of women seeking PAC, which is often a signal indicating the presence of unsafe abortion use. These findings from Ipas’s initial field assessment suggested a need and demand for safe abortion services among Rohingya refugee women.

### Rapid roll-out of abortion services for Rohingya refugees

Following the initial field assessment indicating an urgent need for abortion services, Ipas, together with their national partners Reproductive Health Services Training and Education Program and the Bangladesh Association for Prevention of Septic Abortion [26], quickly appealed to the UNFPA and the Bangladesh Ministry of Health and Family Welfare (MOHFW) to let them start training service providers, and preparing facilities and partners to introduce MR and PAC services in accordance with the national standards and guidelines for SRH service provision in Bangladesh. Gathering support from local colleagues who had worked with Ipas and were currently at UNFPA, government partners were able to begin to garner support for humanitarian MR services at the national level.

Less than one month after the initial surge of Rohingya refugees, Ipas Bangladesh, with the support of UNFPA and Research, Training and Management International, had established MR and PAC services in 8 strategically located facilities serving refugees in the Cox’s Bazaar region. The implementation included the following components: baseline assessment; establishing partner and key stakeholder engagement and approval of the Bangladesh government; supply of medication and vacuum aspiration commodities available in the MOHFW and MISP procurement systems; supply of equipment for facility readiness; strengthening the capacity of health care providers; and strengthening of referral sites for severe complications of unsafe abortions. Although Ipas staff were not working in the Cox’s Bazar district, they were able to mobilize quickly due to longstanding relationships with UNFPA and the MOHFW. Ipas also self-funded the initial assessment and training activities using a small amount of internal rapid response funding. Capacity building and training strategies were developed to cause minimal disruption to service provision. The duration of the formal trainings was adjusted, and time was prioritized for practicum sessions on pelvic models and actual women seeking care to ensure adequate uptake of skill and knowledge of the procedures. The approaches to training and site strengthening addressed stigma, values and attitudes about MR through values clarification and attitude transformation exercises [27]. Following the training, clinical trainers and project staff provided onsite programmatic support to ensure enough logistic supply and clinical mentoring to newly trained providers to assess for competency and confidence with providing MR and PAC services.

Reduced from an original training time of two weeks, the team conducted their first 3-day training in October 2017, training government and NGO physicians and midwives from eight facilities in the use of MVA at a Bangladeshi District Hospital. MR with medication training was provided for two days separately on-site to ensure adequate coverage at the sites for the provision of sexual and reproductive health care. During October–December the model continued to expand services to additional providers and facilities, conducting 4 trainings, in total, during the first three months of the emergency. A combination of focused on-site training and a longer session of frequent supervisory visits was developed to support the increased demands and short supply of health workers in the crisis setting. In February of 2018, the project received support from an additional donor to facilitate additional training and support for 8 more service delivery points, again with health care workers from both government and NGO-supported facilities. By June 2019, almost 300 mostly midlevel health care workers have received clinical training and technical updates on abortion care delivery using guidelines for MR and PAC used in Bangladesh. Today, MR is included in the MISP for primary health care facilities in the Rohingya camps as well as the Global Health Cluster of the World Health Organization in charge of the overall coordination of activities.

### Establishing a “new normal” of MR services for Rohingya refugees

Tentative at first, leadership from UNFPA eventually became more solidified as trainings continued and service provision was introduced. Although UNFPA support grew over time, the willingness of the staff in charge of this process to stand behind these efforts cannot be underestimated. Their leadership in a difficult policy environment was key to the introduction and expansion of these efforts even as they were being attacked by conservative press and social media [26]. The strong and supportive technical leadership of the UNFPA representatives and their willingness to push ahead despite the obstacles, was key to the successful roll-out of MR services in the Rohingya refugee camps.

Stakeholders identified several factors as facilitating the launch and service integration of safe abortion care in Cox’s Bazar. Firstly, historical development partners in Bangladesh had existing ties with national partners who brought local interests to the group and provided valuable insight and opportunity to UNFPA operations. Secondly and instrumentally, MOHFW leadership from within Bangladesh allowed for the introduction and promotion of equitable services for refugees, providing care that was already well-established in the country. With strong comprehensive SRH standards and guidelines, which included MR, already in place throughout Bangladesh, there was little need to develop new standards of care for humanitarian settings. Additionally, international NGO authorization to operate in Bangladesh is time-consuming and tightly controlled by the government; organizations, including several humanitarian organizations, were delayed from setting up initial operations if they were not already providing development assistance in Bangladesh at the time the emergency began. Some larger humanitarian organizations receiving U.S. global health assistance were excluded from operating any abortion-related services during this emergency, which allowed for existing development partners with long-established service histories in Bangladesh to provide a context-driven, grass-roots technical leadership and take up positions in the technical leadership group operating in Cox’s Bazar, positions that would normally have been reserved for international humanitarian agencies.

While the environment for scaling up abortion services proved positive for Rohingya refugees, the national and NGO policies for this service remains unclear and at risk. According to SRH standards and guidelines in Bangladesh, only trained physicians can provide MR up to 12 weeks amenorrhea; midlevel providers, namely paramedics, nurses, and female welfare volunteers, have government authorization to provide early abortion care with both MVA and medication up to ten weeks amenorrhea. There continue to be policy restrictions in Bangladesh limiting the time period to ten weeks for the provision of abortion care by non-physician providers, even after publication of World Health Organization research and guidance supporting no difference in the safety and efficacy of abortions provided by midlevel providers when compared to doctors [28, 29]. Until very recently, midwifery training was nonexistent in Bangladesh; fortunately, the graduation of the first group of midwives trained by UNFPA to provide MR coincided with the Rohingya refugee influx. In an unusual policy decision deemed necessary by the health worker shortage in the camps, the graduating class of midwives was authorized to provide MR and received additional training to improve their knowledge of these services. The remoteness of the region contributed to making health worker shortages even more acute. Midwives who were willing were quickly hired or deployed by UNFPA to camp facilities and they proved to be amenable to skill-building and providing abortion services, having recently completed their coursework on the topic [30, 31]. Through this process, new norms were established with government leadership and the UNFPA-trained work force of midwives. Humanitarian and development partners recognized the growing numbers of refugee women seeking care in their facilities and came to accept and support the introduction of MR as a basic part of their health services. When asked about her feelings about providing MR, one midwife told Ipas staff, “We are human beings and they are human beings, they should receive all the services available”.

### Lessons for providing abortion care in future humanitarian settings

The experience of providing abortion services to Rohingya refugee women in Bangladesh offers some critical lessons for others seeking to uphold the equity of women’s health care in other humanitarian settings. To make progress in this area, several opportunities need to align, and organizations must be willing to adapt to a rapidly changing and erratic humanitarian setting. Being willing to adapt or having already adapted training modalities that allow for on-site and shorter residential training is essential. Having training partners committed to and skilled in facilitation is also necessary for success. Finally, efforts should be made to choose health facilities and health workers wisely prior to training potentially using screening criteria to select for motivation and commitment. Trainees should be able to commit to using their skills to perform a service that may be challenging to start, can lack prioritization, and can be stigmatized by colleagues. Not everyone is prepared to face these challenges.

Even in a country like Bangladesh, with a progressive policy allowing abortion access and a host country MOHFW involved in the provision of refugee care, challenges to equitable SRH for refugee women remain. Although safe abortion service utilization continues to increase in refugee camp facilities that provide such services, the service is new and knowledge about MR is inconsistent. While many agencies are involved in awareness-raising activities through dedicated community health workers, most organizations lack the will, skills and materials to provide accurate reliable information on the availability of MR services.

Another area for potential improvement in this area is in the integration of SRH and protection services, usually the first line of referral for survivors of sexual violence and an area of great need for Rohingya refugees [4, 5]. Unfortunately, the clinical management of rape guidance produced by the World Health Organization and the United Nations High Commission for Refugees, most often used in these settings, focuses only on administration of emergency contraception through 5 days after intercourse, and post-exposure prophylaxis for the prevention of HIV infection and the prevention of sexually transmitted infections [32]. Little attention has been given to unintended pregnancy as sequelae of rape in humanitarian settings. Since most humanitarian partners lack the expertise and willingness to provide abortion in the event of an unwanted pregnancy, it seems likely that this service is not offered [15].

## Conclusions: from one to many – making safe abortion a reality for all refugees

As most of the refugees who fled Myanmar in Bangladesh approach two years in refugee camps in Cox’s Bazar, Ipas is now supporting MR services in 37 health facilities in the district. Although significant progress has been made in MR service availability and quality, many NGOs continue to cause delays to appropriate care because they are not able or willing to provide MR, or care for complications of unsafe abortions, due to perceived or real organizational or national prohibitions. This service began with only 8 facilities in 2017, out of more than 180 health facilities serving over 1,000,000 people. By mid-2019, over 300 health care workers from 37 health facilities had attended clinical training in MR, PAC, and contraceptive provision. More than 8000 Rohingya refugee women and girls have received abortion care and more than three-quarters of these were MR procedures, or safe and legal abortions; more than 26,000 women and girls have received contraception at these facilities. This progress is promising, but the unmet need for these services is still tremendous.

This project shows that the demand for abortion care exists among refugees. It also illustrates that these needs could have been easily overlooked in the complex environment of competing priorities during an emergency, unless championed by technical experts. When services were made available, with relative ease and institutional support, women sought assistance, saving them from complications of unsafe abortions and potentially nonexistent or poor-quality PAC services.

As the world’s numbers of conflicts and disasters and those affected by them continue to grow, it is certain that gains in contributing to the Sustainable Development Goals will need to come from fragile areas, emergencies, and protracted crises where 60% of preventable maternal mortality occurs. Unsafe abortion is among the five main causes of maternal deaths. Preventing those deaths by providing access to safe abortion care for unintended pregnancies, particularly for those most vulnerable to them, is an essential solution. The early introduction of abortion services in Bangladesh for Rohingya refugees proves that this is possible. What remains is the leadership and political will to make it happen in all humanitarian settings.

## Data Availability

The datasets used and/or analyzed during the current study are available from the corresponding author on reasonable request.

## Abbreviations

IAFM: Inter-Agency Field Manual
IAWG: Inter-Agency Working Group on Reproductive Health in Crisis
MVA: manual vacuum aspiration
MISP: Minimum Initial Service Package
MOHFW: Ministry of Health and Family Welfare
MR: Menstrual regulation
MSF: Médecins Sans Frontières
NGO: Non-Governmental Organization
PAC: Postabortion care
SRH: Sexual and Reproductive Health
UN: United Nations
UNFPA: United Nations Population Fund
